# Sunburn in Grapes: A Review

**DOI:** 10.3389/fpls.2020.604691

**Published:** 2021-01-08

**Authors:** Joanna M. Gambetta, Bruno P. Holzapfel, Manfred Stoll, Matthias Friedel

**Affiliations:** ^1^School of Agricultural and Wine Sciences, National Wine and Grape Industry Centre, Charles Sturt University, Wagga Wagga, NSW, Australia; ^2^Department of Primary Industries, National Wine and Grape Industry Centre, Wagga Wagga, NSW, Australia; ^3^Department of General and Organic Viticulture, Hochschule Geisenheim University, Geisenheim, Germany

**Keywords:** sunburn browning, sunburn necrosis, ROS, photooxidation, antioxidants, mitigation

## Abstract

Sunburn is a physiological disorder that affects the visual and organoleptic properties of grapes. The appearance of brown and necrotic spots severely affects the commercial value of the fruit, and in extreme cases, significantly decreases yield. Depending on the severity of the damage and the driving factors, sunburn on grapes can be classified as sunburn browning (SB) or as sunburn necrosis (SN). Sunburn results from a combination of excessive photosynthetically active radiation (PAR) and UV radiation and temperature that can be exacerbated by other stress factors such as water deficit. Fruit respond to these by activating antioxidant defense mechanisms, *de novo* synthesis of optical screening compounds and heat-shock proteins as well as through morphological adaptation. This review summarizes the current knowledge on sunburn in grapes and compares it with relevant literature on other fruits. It also discusses the different factors affecting the appearance and degree of sunburn, as well as the biochemical response of grapes to this phenomenon and different potential mitigation strategies. This review proposes further directions for research into sunburn in grapes.

## Introduction

**Figure 1:**
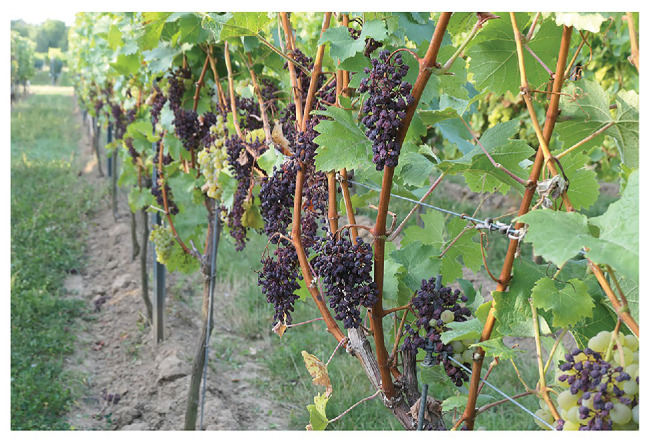
Sunburn necrosis (SN) of Bacchus, a highly susceptible grape variety in the field after bunch zone defoliation.

Sunburn occurs in the field as the result of a combination of high-light intensities, high temperature, and UV radiation (Rustioni et al., 2014). Incidence and severity of the damage depend on a complex interplay of these factors together with the biochemical, physiological, and morphological condition of the berry, all of which are a function of the phenological stage, cultivar and adaptation to meteorological conditions. Symptoms range from the appearance of brown or necrotic spots on the epidermis of grapes to the complete desiccation of the berries. Sunburn represents a serious defect in table grapes, as browning strongly decreases the market value of the crop (United States Department of Agriculture, 1999; Suehiro et al., 2014), and causes significant losses in quality and yield of wine grapes (Figure 1). In Australia, sunburn affects 5–15% of the total wine grape production (Greer et al., 2006), and observations in Chile indicate that up to 40% of bunches can show sunburn damage in sensitive varieties like Muscat (Calderon-Orellana et al., 2018). In other crops such as blueberries (10% value loss in both Washington and Oregon in 2015; Yang et al., 2019), apples (10–50% crop losses reported in South Africa; Wand et al., 2006), pomegranates (30% crop loss; Melgarejo et al., 2004), and red bell peppers (12–36% loss; Barber and Sharpe, 1971; Rylski and Spigelman, 1986) the economic damage caused by sunburn can sometimes be more severe than in grapevines. Depending on the severity of the damage, grapes for wine production in Australia can be downgraded from an A-grade quality to a C‐ or D-grade with a consequent economic loss of ~50% of the crop’s value (Gambetta et al., 2019a). In European viticultural regions, sunburn symptoms occur less frequently and do not necessarily lead to a downgrading of the fruit. Nevertheless, historical records show an increasing frequency of years with significant sunburn damages for German wine-producing regions (1892, 1930, 1947, 1966, 1973, 1998, 2007, 2012, and 2019; Zschokke, 1930; Mohr and Düring, 2000; Schultz, 2007; Stoll and Schultz, 2013, 2020). In France, this phenomenon has been mainly attributed to the higher frequency and intensity of heatwaves, in particular those experienced in 1994, 1998, 2003, 2015, and more recently, 2019 (INRA, 2003; Aubert, 2015; Tupinier, 2019). In Champagne, 5–15% of yield was lost for the years 1994 and 1998 due to sunburn (Mohr and Düring, 2000).

Given the projected increase in air temperatures, the higher frequency and intensity of heatwaves and the phenomenon of global brightening (Wild, 2016), sunburn damage to grapes will inevitably increase in the coming decades. This urges a better understanding and classification of this phenomenon, as well as the reconsideration of canopy management and trellis systems, row orientation, and other preventive measures to protect future berry crops from sunburn. The aim of this review is to provide an accurate description of sunburn, suggest a standard terminology, and give an overview of the factors causing sunburn in grapes and influencing its incidence and severity. The main physiological and chemical changes resulting from grape exposure to high-light and heat stress along with their consequences for grape quality will be discussed together with applicable protective measures. Further fields of research will be identified based on the current state of research.

## Description of the Phenomenon

Sunburn damages berry epidermal tissue at several levels. At the epicuticular level, sunburn causes degradation of the crystalline structure of the waxes into amorphous masses, which leads to a higher water permeability and dehydration, as well as to changes to its visual appearance (Bondada and Keller, 2012). At the epidermal level, it leads to the destruction of chlorophyll (and loss of green coloration) and causes a loss of cell compartmentalization, which exposes polyphenolic compounds to polyphenol oxidases (POX). The oxidation of polyphenols leads to the typical browning of the skin (Olivares-Soto et al., 2020). Oxidation has been observed even in the sub-epidermal layers of the fruit where damage has been reported as far as the seeds (Zschokke, 1930). Similarly, Greer and La Borde (2006) observed that brown sunburn lesions increased in size and depth over time, although they did not report the final depth of browning. This brown coloration has also been attributed to cell death in the epidermal layers of the exocarp (Greer et al., 2006; Nuzzo et al., 2009; Bondada and Keller, 2012) as evidenced by a higher electrical conductivity (and electrolyte leakage) in the peels of affected fruit (Schrader et al., 2001).

Considering the toll sunburn has on grapevine yield and quality, it is surprising that no consistent description of the phenomenon has been adopted in viticulture yet. Consequently, the phenomena described as severe sunburn damage in Chilean vineyards (Calderon-Orellana et al., 2018) might not even be recognized as sunburn under central European conditions, where the term sunburn includes some degree of shriveling. The only reports differentiating sunburn phenomena in grapes we are aware of were made by Krasnow et al. (2010), reporting sunburn browning (SB), sunburn cracking, and poor color development of red varieties as symptoms, and 80 years earlier by Zschokke (1930). Zschokke (1930) reported different levels of sunburn damage: sunburn spots on the berry skin, complete or partial shriveling of berries, and damages to the rachis and consequent shriveling of entire sections of the bunch. He also reported poor color development of red varieties attained by sunburn. This stands in contrast to other horticultural crops like apples, where the symptoms and driving factors of three different types of sunburn phenomena – SB, sunburn necrosis (SN), and photooxidative sunburn (PS) – have been accurately described (Racskó and Schrader, 2012).

**Figure 2:**
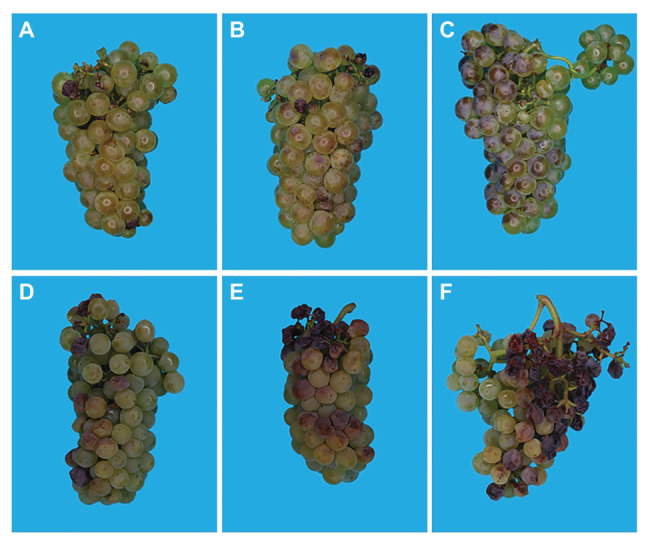
Images of Chardonnay bunches with increasing degrees of sunburn browning (SB; **A–C**, 0–51%) and SN (**D–F**, 12–32%) damage. Pictures were taken at harvest (~22°Brix) in Orange, Australia.

Sunburn browning is the result of a combination of both high light and high temperature, and is observed mainly after véraison (Schrader et al., 2001). It is considered a sub-lethal form of damage that causes the appearance of yellow, brown, or bronze spots on the sun-exposed side of the fruit (Figures 2A–C; Schrader et al., 2009). In white grapes, sunburn causes brown lesions on the surface of the berry, and in red berries, SB affects anthocyanin biosynthesis and manifests as poor color development and bleached spots (Greer et al., 2006; Bondada and Keller, 2012; Bondada, 2019).

**Figure 3:**
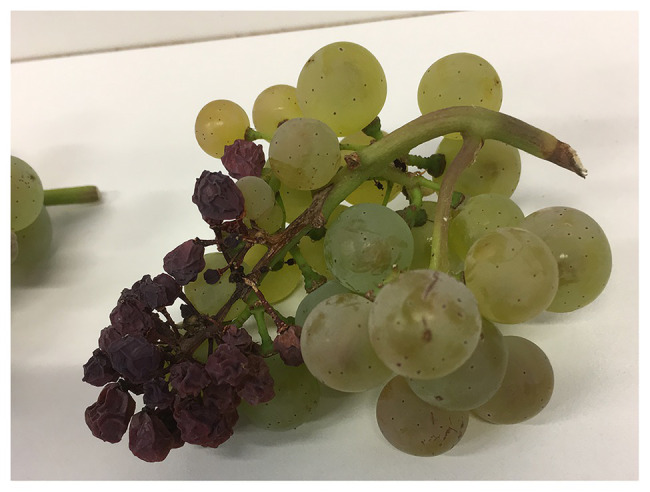
Rachis damage caused by SN in Riesling. 47% of berries were damaged due to a sunburn event occurring on July 25, 2019. Picture was taken on September 30, 2019, at 19.5°Brix in Geisenheim, Germany.

Sunburn necrosis is mainly a function of high temperature, and requires significantly higher temperature levels than those necessary for SB to occur. SN can be considered a lethal damage appreciated by the appearance of dark brown or black necrotic spots on the fruit’s surface, where severe cases can lead to berry cracking and shriveling (Figures 2D–F; Barber and Sharpe, 1971; Schrader et al., 2003; Krasnow et al., 2010). SN causes serious changes in the cuticular, epidermal, and sub-epidermal tissues ultimately destroying the integrity of cell membranes (Schrader et al., 2001). Pre-véraison SN leads to shriveling of entire berries, affects parts of the rachis and even entire bunches (Figure 3), and leads to considerable yield losses.

Photooxidative sunburn is caused exclusively by an excessive amount of photosynthetically active radiation (PAR; Felicetti and Schrader, 2009) and manifests as bleached pigments and, in severe cases, necrosis. There are no records of PS in grapes in the field to date.

## Environmental Factors Affecting Sunburn Development

### Light as an Inducing Factor

Solar radiation can be divided into UV (UV-A, 400–315 nm and UV-B, 315–280 nm), visible (400–780 nm), which includes PAR (400–700 nm), and infrared radiation (IR, >780 nm). The intensity of these depends on altitude, latitude, season, time of day, and cloud coverage (McKenzie et al., 2003). Light acts both as a source of heat (section Ambient and Fruit Surface Temperature) and as the driver of photochemical and oxidative reactions in the berry, where photooxidation plays a central role in the development of SB symptoms. Regardless of the temperature, neither SN nor SB is observed in well-shaded bunches in the field (Rustioni et al., 2014).

An excessive amount of light promotes the production of triplet chlorophyll (^3^Chl*) and reactive oxygen species [ROS; singlet oxygen (^1^O_2_), superoxide anion (O_2_^−^), hydrogen peroxide (H_2_O_2_), and hydroxyl radical (HO·)], all promotors of oxidative stress in the plant’s and fruit’s photosystems. Of these, HO· is the ROS with the shortest half-life and highest phytotoxicity. It can be generated from H_2_O_2_ in the Fe-S center of photosystem I (PSI) through a process termed the Fenton reaction, which is catalyzed by metal ions such as Fe^2+^, and peroxidases. Although ROS are normally present in non-stressed cells, stress conditions lead to a drastic increase of these highly reactive molecules and a reduction of photosynthetic CO_2_ fixation, leading to excess excitation energy captured by PSI and PSII (measured as the maximum quantum yield of chlorophyll fluorescence, *F*_v_/*F*_m_; Mittler et al., 2004; Glenn and Yuri, 2013). Stress conditions like high temperature or drought have been associated with increased ROS production (Carvalho et al., 2016). The plant can then either tolerate and adapt to the new levels of ROS or suffer some form of damage.

Photosynthetically active radiation and UV are the two main components of light involved in sunburn development. Exposure to high PAR levels decreases *F*_v_/*F*_m_ of the exposed tissue, and as a consequence, non-photochemical quenching (NPQ) of PSII increases in an attempt to protect the photosystems. If PAR overexposure continues, NPQ becomes photoinhibited and sunburn damage ensues (Glenn and Yuri, 2013; Rustioni et al., 2015). UV is a high-energy form of radiation, which induces mutations if absorbed by DNA, inhibits electron transport, and collapses membrane integrity (Jenkins, 2009). Response to UV depends on the dose, duration, and wavelength the organ is exposed to. High fluence rates combined with short wavelengths cause stress responses and lead to necrosis whilst low rates initiate regulatory responses that promote the production of photoprotective compounds (Kolb et al., 2003; Jenkins, 2009; Pastore et al., 2013). Despite having relatively low average temperatures, areas like New Zealand and Chile report high incidences of sunburn in grapes and apples, most probably due to their high UV index (Hofmann et al., 2006; Schrader et al., 2008). Locations in the southern hemisphere receive on average 12–15% more UV radiation than similar locations in the northern hemisphere with this difference increasing as latitude decreases (Gregan et al., 2012). Studies on the effect of PAR and UV have demonstrated that the interaction between them results in greater changes in *F*_v_/*F*_m_ and fruit composition when compared to each separate factor alone. The UV × PAR interaction plays a key role in the initiation of sunburn damage, although PAR plays a greater role in the degradation of the berry’s photosystems (Glenn and Yuri, 2013; Joubert et al., 2016). An influence of IR-radiation on the development of sunburn has not been reported in fruits yet.

### Ambient and Fruit Surface Temperature

Temperature is a major source of abiotic stress that affects many physiological responses at the plant and fruit level. Although there is no specific molecule that acts as a thermosensor, fruits possess a diverse intracellular signaling mechanism that is activated in response to heat. Thermal stress has amongst its main targets the photosynthetic apparatus, which consequently undergoes a series of reversible changes to cope with heat, although when the heat is excessive, the photosystems can be severely and irreversibly damaged (Araújo et al., 2018). High temperature induces an imbalance between light energy absorption and usage impairing electron transport activity. Consequently, fruit respiratory mechanisms are altered and the higher level of anaerobic respiration caused by higher temperatures induces the accumulation of ROS (Jiang et al., 2015). Chloroplasts themselves can be damaged or degraded by heat stress (Hu et al., 2020). Thermal stress can cause membrane destabilization, protein denaturation, and berry pericarp cell death. Experiments have demonstrated that high temperatures bring cell death forward in Shiraz by ~9 days (Bonada et al., 2013). Furthermore, elevated heat alters the regulation of major metabolic pathways and the expression of genes involved in all levels of plant physiology (Mittler et al., 2012). In grapevine, heat events (>30°C) have deep consequences for berry growth and composition (Hale and Buttrose, 1974; Pastore et al., 2017).

Although a purely PS has been induced in grapes under laboratory conditions (Rustioni et al., 2020), the prevailing type of damage in vineyards results from the combination of high light and high temperatures. Very little to no damage occurred when greenhouse-grown berries were exposed to high light intensities at low-moderate temperatures (25–30°C). However, when the temperature was increased to 38°C, damage of ripe Semillon berries was observed even at low light intensities, and was devastating at high light intensities with 94% of bunches affected (Hulands et al., 2014).

**Figure 4:**
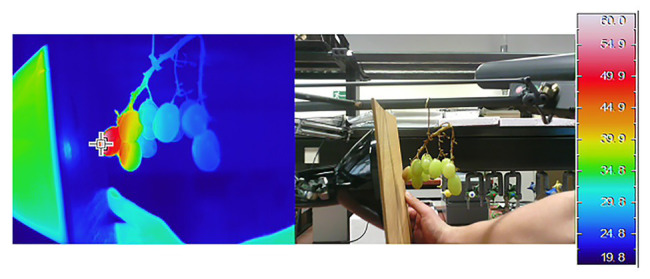
Infrared and RGB pictures of grape berries heated with an infrared heat emitter. The temperature gradients induced by IR heating allow for the determination of threshold temperature for the appearance of necrotic spots. In this example, detached ripe Sultana grapes (19.3°Brix) suffered SN damage at 52°C.

Berry temperature is a function of air temperature and radiative heat transfer – there is a linear relationship between temperature and light absorbed by the berry tissue (Hulands et al., 2014) which makes it very difficult to separate the effect of these two factors, especially when conducting vineyard studies. Direct exposure to the sun increases fruit surface temperature (FST) by as much as 12–15°C above air temperature on the berry’s sun-exposed side (Smart and Sinclair, 1976; Spayd et al., 2002). Consequently, FST can vary widely according to bunch location in the canopy and level of solar exposure (Spayd et al., 2002). FST is also modulated by wind velocity, berry color, and bunch compactness (Dry, 2009). In the field, exposed dark berries can have temperatures up to 5°C higher than white berries (Spayd et al., 2002). Sunburn of different crops has been observed between the thresholds of 45–49°C (Schrader et al., 2008; Genovese et al., 2010; Yang, 2018), values that are rarely reached in the field without radiative heat transfer. This implies that FST is more relevant for sunburn induction than ambient temperature. FST also modulates the type of damage observed; when FST of apples reaches 52 ± 1°C SN occurs within 10 min whilst SB occurs when FST of sun-exposed apples reaches 46–49°C for an hour (Schrader et al., 2003). Own experiments have shown the occurrence of SN in detached white table grape berries after 15 min of exposure to 52°C in the absence of solar radiation (Figure 4).

## Biochemical Response of Grapes to Light and Heat Stress

**Figure 5:**
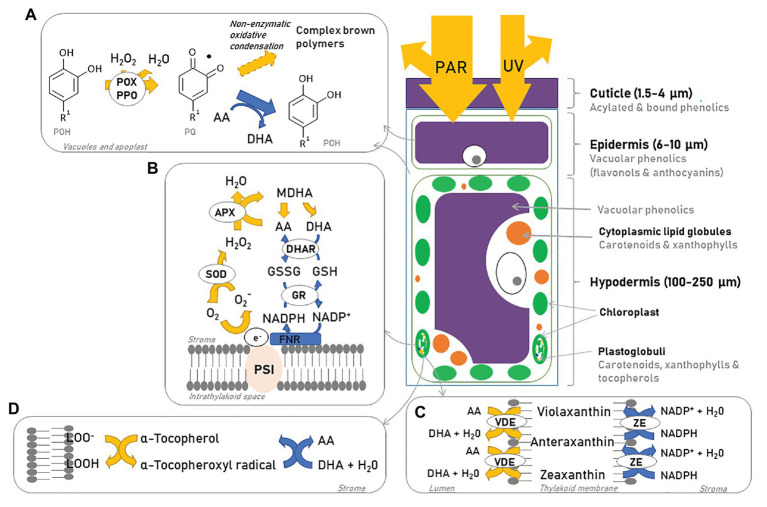
Epidermal cell, photoprotection, and reactive oxygen species (ROS) scavenging mechanisms. As photosynthetically active radiation (PAR) and UV light reach the berry, part of these forms of radiation are reflected by the cuticle. Vacuolar phenolics **(A)** act as a screen helping to reduce the amount of incident light further penetrating the cell and help mitigate part of the ROS formed through the formation of oxidized phenolic forms and complex brown polymers (if ascorbic acid is absent). If light penetrates further into the hypodermis, the chloroplasts and mitochondria become the main target of radiation. The water-water cycle **(B)**, non-photochemical quenching (NPQ; **C**), and tocopherol **(D)** are used to remove ROS and prevent damage to the photosystems. AA, ascorbic acid; DHA, dehydroascorbate; MDHA, monodehydroascorbate; DHAR, dehydroascorbate reductase; APX, ascorbate peroxidase; SOD, superoxide dismutase; GSSG, glutathione disulfide; GSH, glutathione; GR, glutathione reductase; PSI, photosystem I; POX, polyphenol oxidase; PPO, polyphenol peroxidase; POH, polyphenol; PQ, oxidized phenol; VDE, violaxanthin de-epoxidase; ZE, zeaxanthin epoxidase. Based on Solovchenko and Merzlyak (2008).

Grapes regulate a number of physiological and biochemical processes as a response to a higher light and temperature environment to minimize damage to their photosynthetic system. Plants need to maintain fruit photosynthesis, which is important for fruit development, in particular in green berries. Protection from direct and ROS-mediated damage is achieved by dissipating the excess energy as heat through NPQ (Müller et al., 2001) and oxidative damage is alleviated *via* antioxidant enzymes, soluble antioxidants, and ROS scavengers (Figure 5; Carvalho et al., 2016).

### Enzymatic Activity and Antioxidants

**Figure 6:**
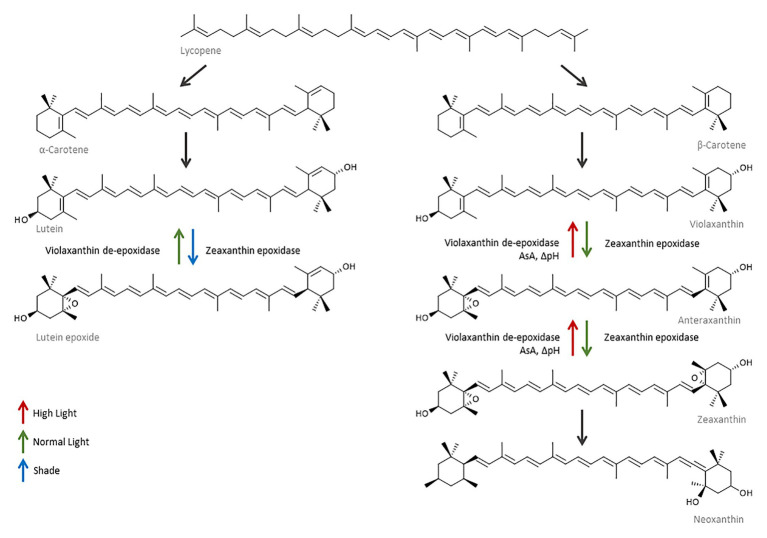
Carotenoid synthesis is up-regulated in response to changes in the light environment. As a consequence of higher light, α‐ and β-carotene are synthesized from lycopene and used to produce more lutein and violaxanthin. The violaxanthin cycle is rapidly induced in response to high light, and violaxanthin is epoxidized first to anteraxanthin and then to zeaxanthin. Violaxanthin de-epoxidase (VDE) requires ascorbate (AsA) and a pH gradient to catalyze the reaction. In the absence of ascorbate, zeaxanthin is converted to neoxanthin. The lutein epoxide cycle converts lutein epoxide into lutein and is induced when tissues move from a shade to normal light situation or under prolonged high light stress.

As a consequence of photooxidative and thermal stress, the activity of a suite of ROS-scavenging enzymes [e.g., ascorbate peroxidase (APX), ascorbate-glutathione cycle enzymes, superoxide dismutase (SOD), and catalase] increases, the production of antioxidant metabolites (e.g., ascorbate, glutathione, and α-tocopherol) is up-regulated and their reduction state increased (Thompson et al., 1987; Ma and Cheng, 2003; Jenkins, 2009). Ascorbate and glutathione are key water-soluble antioxidants located in the chloroplasts and the main objective of the ascorbate-glutathione cycle is to detoxify ROS *via* photoreduction of H_2_O_2_ into water and oxygen (Figure 5B; Ma and Cheng, 2003). The upregulation of the ascorbate-glutathione cycle is synchronized with the xanthophyll cycle (section Carotenoids) – the de-epoxidation of violaxanthin uses reduced ascorbate as reductant (Figures 5C, 6), which then regenerates *via* the ascorbate-glutathione cycle. Ascorbate deficiency can limit the de-epoxidation of violaxanthin and lower NPQ by limiting violaxanthin de-epoxygenase (VDE) activity (Müller-Moulé et al., 2002; Ma and Cheng, 2003). Ascorbate also plays a role in the Mehler-peroxidase reaction (also known as the water-water cycle) used by PSI to reduce ROS (Figure 5B). Therefore, the Mehler-peroxidase reaction competes with VDE for ascorbate but might also be involved in creating a sufficient pH gradient to activate VDE (Müller-Moulé et al., 2002). α-Tocopherol is a hydrophobic antioxidant associated with membranes. It quenches ^1^O_2_ and reacts with superoxide and lipid peroxy radicals to form tocopherol semiquinone and prevent lipid peroxidation (Figure 5C). Tocopherol semiquinones can be reduced by ascorbate, which is oxidized to dehydroascorbic acid (DHA) and later regenerated in the presence of glutathione (Thompson et al., 1987; Havaux, 2014).

Once oxidation processes have been initiated, ascorbate can suppress the complete oxidation of phenolic compounds by POX and polyphenol peroxidase (PPO) that lead to enzymatic browning. The blackening of the epidermis after high light exposure results from the polymerization of vacuolar phenolics as the result of the penetration of H_2_O_2_ into vacuoles of epidermal cells and the activity of POX and PPO. However, POX can help scavenge H_2_O_2_ by using flavonols as electron donors (Figure 5A; Yamasaki et al., 1997). When this reaction is coupled to the ascorbate-glutathione cycle, ascorbate reduces the primary oxidized product of phenolics to their parent compounds and produces water and DHA, thus inhibiting the formation of degradation products and more O_2_^−^ and H_2_O_2_ (Yamasaki et al., 1997; Makris and Rossiter, 2002; Hernández et al., 2009). In the absence of ascorbate, polymerization products of flavonoids and other polyphenols may be irreversibly generated.

### Pigments and Photoprotective Compounds

Plants possess multiple photoreceptors that are responsible for the activation of various signal transduction cascades that regulate light-dependent responses and related gene expression. These include the phytochrome superfamily, which consists of photoreceptors absorbing red/far-red light, cryptochromes (blue, green, and UV-A), phototropins (UV-A/blue-light), and UV-B photoreceptors (photoreceptor UV RESISTANCE LOCUS 8, UVR-8). After exposure to PAR or UV, these receptors up-regulate the expression of genes coding for photoprotective molecules such as carotenoids and flavonoids to protect the berry’s DNA and photosynthetic apparatus from further damage.

#### Carotenoids

Carotenoid accumulation plays an important role in the photoprotection of grape berries; they are efficient antioxidants capable of scavenging ^1^O_2_* and peroxyl radicals, quenching ^3^Chl* generated during photooxidation processes, and possess the ability to screen light in the blue-green (450–570 nm) and UV part of the spectrum. They are also capable of modifying membrane fluidity, thereby increasing its thermostability and protecting it from lipid peroxidation (Solovchenko and Merzlyak, 2008; Joubert et al., 2016). The xanthophyll cycle is one of the most important antioxidant systems in grapes and constitutes one of the main modes of action of NPQ (Figures 5C, 6). It is a rapidly induced and rapidly reversible mechanism. In the green berry stage, the activation and interconversion of the xanthophylls violaxanthin (V), anteraxanthin (A), and zeaxanthin (Z) under excessive light conditions takes only minutes and helps quench ^1^O_2_ and dissipate excess excitation energy of ^3^Chl* as heat. Consequently, V is first de-epoxidized to A and then to Z in a reaction mediated by VDE and catalyzed by ascorbate. A second xanthophyll cycle constituted by lutein and lutein epoxide works in a similar way to regulate NPQ, but has a slower relaxation rate and is thought to aid in situations of prolonged stress (Figure 6; Joubert et al., 2016). At noon, almost all the xanthophyll cycle pool in sun-exposed peel is present as A + Z indicating that the xanthophyll cycle is operating at full capacity and that the pool size may become limiting in a higher stress situation, for example, at elevated temperatures and/or if the stress continues over a sustained period of time (Ma and Cheng, 2003). Zeaxanthin and lutein may also have a direct role in the protection of the thylakoid membrane, acting as antioxidants against lipid peroxidation by ROS (Müller et al., 2001). β-Carotene acts as a direct precursor to V, but also as an accessory pigment located in P680 reaction centers, where it protects the photosynthetic apparatus by scavenging ^1^O_2_* and quenching ^3^Chl* (Felicetti and Schrader, 2009). Neoxanthin has also been implicated in energy-dependent quenching (Müller et al., 2001). Whether xanthophylls are directly or indirectly involved in the de-excitation of ^3^Chl*, is still unknown.

#### Phenolic Compounds

Phenolic compounds include the flavonoids (flavonols, flavan-3-ols, and anthocyanins) and the non-flavonoids (stilbenes, hydroxycinnamic acids, and hydroxybenzoic acids and their derivatives). Their accumulation in the berry skin is strongly regulated by changes in the fruit environment (González et al., 2015). Phenolic compounds accumulate in the berry upper epidermis as well as in the hypodermis and cuticle, where they are used by plant tissues as photoprotectants due to their capacity to absorb and screen PAR and UV light, thereby constituting the plant’s first line of defense against photo stress. They scavenge harmful singlet oxygen and H_2_O_2_, inhibit ROS formation, and quench free radical reaction cascades in lipid peroxidation (Kolb et al., 2003). Polyphenols can also inhibit the Fenton reaction by complexing metals such as ferrous iron (Son and Lee, 2008; Chang et al., 2017).

Flavonols are mainly constituted by quercetin, myricetin, and kaempferol; with lower percentages of laricitrin, isorhamnetin, and syringetin; their profile varying amongst genotypes and grape color. They are present in berries as mono-, di-, and tri-hydroxylated forms and are only accumulated as glycosides. Flavonols have a high extinction coefficient at wavelengths characteristic of UV (Kolb et al., 2003) and their synthesis is strongly and rapidly induced by solar radiation – upon 8 h light exposure, the expression of flavonol synthase (*VvFLS1*) and flavonol glycosyltransferase (*VvGT5* and *VvGT6*) genes increased four-fold on a bunch level (Friedel et al., 2016). Oxidation and polymerization modifies the biological properties of polyphenols, and their polymerized and oxidized forms may further screen light in the PAR range, offering additional protection to chloroplasts (Rustioni, 2017). The antioxidant activity of polyphenols increases with their degree of polymerization up to a mean degree of polymerization of about 10 (Zhou et al., 2014). Polyphenol quinones have also been cited to modulate lipoxygenase activity, preventing membrane damage (Hernández et al., 2009; Ferrandino and Lovisolo, 2014).

Anthocyanins are synthesized in the skin from véraison onwards and include cyanidin, peonidin, delphinidin, petunidin, and malvidin derived pigments. They are involved in the protection against damage by high fluxes of visible radiation. Their maximum absorption range is located in the green range (500–600 nm), which is close to the solar energy peak and coincides with the gap between Chl and carotenoid absorption bands in which light penetrates deeply into plant tissue (Merzlyak and Chivkunova, 2000). A high anthocyanin content increases resistance to Chl photobleaching, as anthocyanins show a higher photostability than Chl (Solovchenko and Merzlyak, 2008).

### Heat Shock Factor

The synthesis of heat shock factor (HSF) and heat shock proteins (HSPs) is considered the first line of defense against thermal stress. They help protect cell membranes from heat damage and lipid peroxidation, and maintain structural and functional proteins’ quality and folding by protecting them from denaturation (Araújo et al., 2018; Hu et al., 2020). Small HSP (smHSP) proteins predominate during heat stress, their levels increase 2000-fold upon heat stress and both smHSP and HSP70 concentrations are positively correlated with sun exposure. The total amount of HSP proteins produced seems to be cultivar related and decreases with grape maturity (Ritenour et al., 2001; Guillaumie et al., 2011).

### Aroma Compounds

Like polyphenols, some volatile compounds such as the terpenes have been recognized as having antioxidant capacity and are capable of quenching excess energy. It is hypothesized that under high-temperature conditions, terpenes act as thermoprotective molecules that stabilize chloroplast membranes (Loreto and Schnitzler, 2010; Joubert et al., 2016).

### Adaptation

#### Biochemical Adaptation

Grapes have the capacity to adapt to changes in microclimatic conditions and thus increase their resistance to sunburn (von Babo, 1840). Acclimation responses depend on the type, dose and duration of the light and thermal stress (Figure 6), to which plants respond by activating stress-signaling pathways that generate, amongst other metabolites, ROS and H_2_O_2_. At low doses, ^1^O_2_* and H_2_O_2_ act as signal transduction molecules and trigger protective mechanisms, while high doses of ROS cause necrosis and cell death (Gechev et al., 2006; Pourcel et al., 2007). Due to their instability, ROS cannot diffuse through membranes (Yamasaki et al., 1997) and must be detoxified *in situ*. Accordingly, the acquisition of photo- and thermo-tolerance seems to be a highly localized process. In apples, fruit rotation has been shown to drastically increase the appearance of sunburn symptoms as shaded fruits are more sensitive to photoinhibition and have lower *F*_v_/*F*_m_ than sun-exposed fruit (Wünsche et al., 2001; Li and Cheng, 2008). Shaded and sun-exposed sides in apples show pronounced differences in skin composition, mainly in the accumulation of phenolics, carotenoids, and anthocyanins, but also chlorophylls, HSPs, and antioxidant enzymes (Ritenour et al., 2001; Merzlyak et al., 2002; Ma and Cheng, 2003). Within the grape cluster, a similar localized accumulation pattern of photoprotectants has been observed: their accumulation varies within a cluster and even within individual berries (Friedel et al., 2012; Pieri et al., 2016).

##### Light

Excessive light induces metabolic responses including the accumulation of antioxidants and of enzymes controlling their redox state (Rustioni et al., 2020). Light exposed berries accumulate higher amounts of ascorbate during berry development when compared to shaded berries (Debolt et al., 2007), and the capability to increase carotenoid concentration in response to light exposure appears as a major photoadaptation mechanism that distinguishes sunburn-susceptible cultivars from more resistant ones (Merzlyak et al., 2002). qRT-PCR analysis of sunburn affected peels of apples showed the upregulation of the genes phytoene synthase (PSY) and phytoene desaturase whilst lycopene β-cyclase and lycopene ɛ-cyclase remained unchanged. PSY converts geranylgeranyl diphosphate into phytoene as the first step of carotenoid biosynthesis, and these genes have been shown to be generally up-regulated by light (Liu et al., 2018). Total carotenoids concentration and the xanthophyll cycle pool are larger in exposed fruit than in shaded grapes when measured before véraison, although some of these differences disappear by harvest (Hickey et al., 2018; Gambetta et al., 2019b). Düring and Davtyan (2002) showed that the relative importance of xanthophyll cycle carotenoids increases during adaptation to high light conditions along with an elevated NPQ.

Results on the effect of sunburn on carotenoid concentration so far have been contrasting due to differences in experimental conditions, ripening stage, and cultivar, but especially, from the choice of sample location in the canopy. Previously acclimated fruit (sun-exposed) appear to react very differently to shaded fruit in these experiments. Some authors report an overall degradation of these compounds as a result of sunburn damage, leading to lower concentrations of Chl, β-carotene, lutein, neoxanthin, and V + A + Z in the peel of injured fruit when compared to non-sunburnt fruit (Torres et al., 2006; Li and Cheng, 2009). Conversely, Felicetti and Schrader (2009) demonstrated a slight increase of V + A + Z and a marked increase in β-carotene in affected fruit, although these results depended on the season. Most authors agree however that the ratio of carotenoids/Chl, V + A + Z/Chl, and Chl a/Chl b increase as a result of the preferential destruction of Chl with sunburn (Ma and Cheng, 2003; Felicetti and Schrader, 2009; Torres et al., 2013) as carotenoids have been reported to be more photostable than Chl, in particular, Chl b (Merzlyak et al., 2002; Felicetti and Schrader, 2009).

Higher light exposure also increases the total amount of flavonoids present in the berry (Pastore et al., 2013; Kok and Bal, 2018; Würz et al., 2018; Brandt et al., 2019; Hickey and Wolf, 2019). UV-B radiation upregulates genes responsible for the synthesis of a range of phenolic compounds including phenylalanine ammonia-lyase (PAL), flavonoid-3'-hydroxylase (F3'H), flavonoid-3',5'-hydroxylase, flavonol synthase (FLS), MYB transcription factor, and UDP-glucosyl transferases (UFGT; Stracke et al., 2010; Czemmel et al., 2012; Pastore et al., 2013). The concentration of quercetin and kaempferol glycosides were up to 10 times higher in sun-exposed Merlot and Pinot Noir berries than in shaded ones (Price et al., 1995; Spayd et al., 2002). UV-B exposure also favors the production of flavonoids with hydroxyl groups on ring B of the flavonoid skeleton, e.g., quercetin glycoside over kaempferol glycoside, thus increasing the potential antioxidant activity of the organ. When exposed to light, a cascade of reactions triggers the synthesis of flavonols and sinapyl derivates. Responsiveness to light induction differs amongst flavonoid classes, with flavonol glycosides being the most responsive ones (Koyama et al., 2012; Reshef et al., 2018). However, adaptive responses to light depend heavily on the stage of ripening (as further discussed in section Developmental Stage). Flavonol production, and the expression of the genes that mediate their synthesis (*VvMYBF1* and *VvFLS1*) peak between flowering and fruit set and decline after véraison, with a later peak at maturity (Downey et al., 2003; Czemmel et al., 2012). After véraison, the expression of anthocyanin-specific genes (*MYBA* and *UFGT*) increases as does anthocyanin accumulation (Czemmel et al., 2012). However, higher light exposure after véraison reduces the expression of genes directly involved in anthocyanin synthesis and transport such as UFGT (Pastore et al., 2013).

##### Temperature

**Figure 7:**
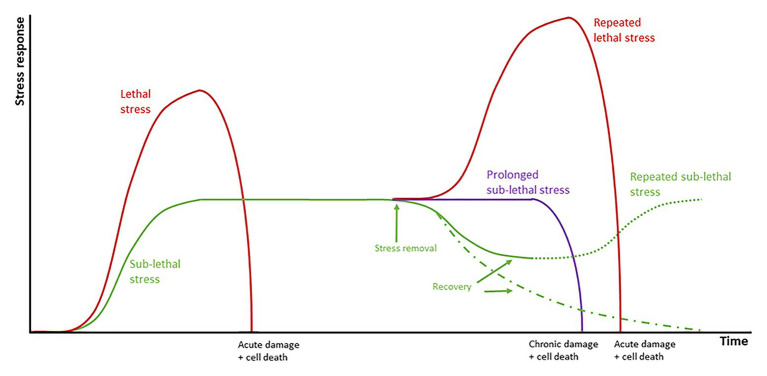
Schematic representation of the responses of grapes to abiotic stress. Plants are initially in a basal state when stress is applied. Stress can be divided into lethal stress (red lines) which lead to acute damage and cell death; and sub-lethal stress (green line) which leads to the activation of a series of stress response mechanisms. Prolonged stress (purple line) leads ultimately to chronic damage and cell death. If sufficient recovery time is allowed, fruit returns to the original basal state (green dotted and dashed lines).

Exposure of tissues to sub-lethal temperatures confers increased transient thermotolerance that protects the plant from a second exposure to lethal temperatures that lead to SN. Thermotolerance is acquired through the accumulation of HSPs, genes encoding detoxification enzymes (e.g., glutathione S-transferase, glutathione reductase, SOD, CAT, peroxidase, and APX), antioxidants (GSH and ascorbate), and regulatory proteins (Lim et al., 2006; Wang and Li, 2006). In experiments on apples conditioned at 38°C, an inverse relationship between the appearance of sunburn symptoms and duration of conditioning was observed, with conditioned apples presenting symptoms at slightly higher temperatures than non-conditioned ones. Conditioning results in less H_2_O_2_ produced, as observed in leaves of whole vines conditioned at 38°C for 12 h and then exposed to 47°C during 2 h (Wang et al., 2009). This thermotolerance, however, is only temporary and degrades under continued stress or if the temperature increases above lethal thresholds (Figure 7; Naschitz et al., 2015).

When plants are subjected to multiple sources of stress simultaneously, antagonistic effects on gene expression are usually observed. Experiments contrasting the effect of high temperature, high light, and combined high light and temperature, have demonstrated that it is this last condition that affects the size of the carotenoid pool the most (Li and Cheng, 2009). As such, high temperatures might slow down or even reverse biochemical acclimation responses by negatively impacting the berry’s antioxidant response. At temperatures above 35°C significantly more H_2_O_2_ is produced whilst the APX pathway, NPQ, APX, and SOD are inhibited (Rocheta et al., 2014). High temperatures affect carotenoid and chlorophyll biosynthesis and degradation by impairing the expression of genes in the carotenoid pathway and increasing the activity of chlorophyllases, peroxidases, and lipoxygenases (Shi et al., 2014). Although sub-lethal temperature increases have a limited impact on flavonoid synthesis (Pastore et al., 2013), temperatures over 30°C decrease overall flavonoid concentration. When the temperature rises above 35°C, enzymatic activity in the flavonoid biosynthetic pathway is impaired and degradation by PPO and POX increases, compromising flavonoid final concentrations (Mori et al., 2007; Mohaved et al., 2016). Anthocyanin accumulation is inhibited at even lower temperatures than flavonols, with the highest accumulation reported at 25°C (Mori et al., 2007). Experiments on apples have demonstrated a significant loss of anthocyanin content in sunburnt apples (~63% loss) together with a reduced level of expression of *MdANR* and *MdFLS* (Liu et al., 2018). Exposure to higher light and temperature also modifies the proportion of non-acylated anthocyanins and the level of B-ring hydroxylation and thus the ratio between di‐ and tri-hydroxylated forms. Tri-substituted anthocyanins have been reported to be more stable at high temperatures and more effective at scavenging free radicals than di-substituted ones, and are more abundant in berries ripened under high-temperature conditions (Mori et al., 2007; Cohen et al., 2012; Koyama et al., 2012) although a field study by Pastore et al. (2013) does not support this theory. Likewise, acylated anthocyanins have been referred to as being more thermostable than their non-acylated counterparts (Tarara and Spayd, 2005) and their relative contribution to the anthocyanin profile is higher in grapes suffering heat stress (Mori et al., 2007).

##### Recovery Periods

Recovery periods are associated with the detoxification and activation of repair mechanisms and are critical to the ability of an organ to adapt to abiotic stress. When allowed recovery periods, the capacity of plants to adapt to different stresses is enhanced when compared to continuous periods of stress (Figure 6). In an experiment comparing constant doses of UV-B (6 h at 0.04 mW cm^−2^) and pulsed doses (6 × 1 h intervals interspersed with 30 min recovery periods), Arabidopsis plants allowed recovery periods produced more photoprotectants; 27% more total flavonols and sinapyl derivates, 38% more kaempferols, and 90% more quercetins (Höll et al., 2019). The authors also demonstrated that the amount by which these compounds increase depends on the duration of the recovery periods, with shorter recovery periods showing almost no differences when compared to plants treated continuously. Kaempferols, quercetins, and sinapyl derivatives required different amounts of recovery time to be expressed, with kaempferol requiring the least (~30 min) and sinapyl derivatives the longest (1.5 h) amounts of time. Similarly, sufficient recovery during low light periods and overnight permitted apples to better withstand sunburn, however, if full recovery did not occur, the damage was accumulated (Glenn and Yuri, 2013). The duration of these recovery periods, and whether a plant is able to recover at all, are contingent on the intensity of the applied stress. When exposed to 25 and 35°C for 5 h, plants allowed a 1 day recovery period recovered their initial photosynthesis rates. However, when the temperature was increased to 40°C, it took plants 2–4 days to recover their initial levels, and when the temperature was increased to 45°C basal levels were not reattained even after 4 days of recovery. It takes temperatures higher than 35°C to cause significant changes to the NPQ capacity of the fruit, however, NPQ returns to basal levels rapidly when sufficient recovery time is allowed, but repeated stress means that this recovery time is prolonged and that irreversible damage can occur (Luo et al., 2011).

##### Morphological Adaptation – Waxes and Epidermis Thickness

**Figure 8:**
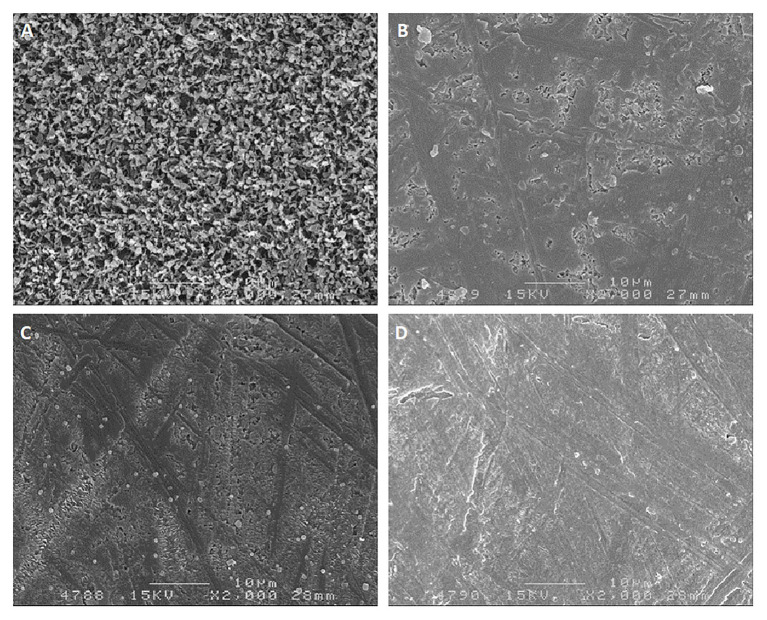
Scanning electron micrographs (×2000 magnification) of epicuticular waxes of Chardonnay grapes. **(A)** Control grapes with no sunburn; **(B)** slight sunburn; **(C)** moderate sunburn; **(D)** severe sunburn (originally from Greer et al., 2006; reprinted with permission from Vitis).

Epicuticular waxes protect the berry against light and heat stress. Although their main function is as transport barriers, they also play a role in protection against PAR and UV radiation by scattering, reflection, and even absorption, thus reducing exposure levels in the underlying tissues (Figure 8). The capacity of this layer to scatter light is dependent on the size, distribution, and orientation of the wax crystals. Plate-like wax crystals reflect and scatter a higher proportion of light than amorphous waxes (Jenks and Ashworth, 1999), while still allowing for transpiration (Muganu et al., 2011). Plate-like wax structures prevail in light-exposed grape berries of several varieties, while berries grown in the shade of the canopy have a higher proportion of amorphous waxes (Muganu et al., 2011). As sunburn symptoms appear, these waxes lose their crystalline structure and become relatively amorphous (Figure 8; Greer et al., 2006).

Sun-exposed berries have a thicker layer of epicuticular wax and overall thicker cell walls than shaded ones (Rosenquist and Morrison, 1989; Muganu et al., 2011; Verdenal et al., 2019), which relates to a higher capacity to reflect light (20–80% of incoming radiation when compared to shaded plants that only reflect 10%; Jenks and Ashworth, 1999). A thicker epidermis also translates into more epidermal layers and increased capacity for anthocyanin and flavonol storage (McDonald et al., 1998; Pastore et al., 2013). Higher accumulation of polyphenols in the cuticle in response to light exposure modifies the cuticle’s optical properties, converting it into a non-uniform filter that absorbs in the UV region (Solovchenko, 2010).

Higher light and thermal stress have also been observed to up-regulate genes involved in lignin precursor synthesis and lignin’s biosynthetic pathway (Cabane et al., 2012; Pastore et al., 2013; Zenoni et al., 2017; Verdenal et al., 2019). Consequently, the peel of sunburnt apples contains higher amounts of lignin than shaded and healthy, sun-exposed organs. Lignification is a mechanism used by plants to increase their resistance to stress, however, the possibility that this increase is also a consequence of cell damage and polyphenol oxidation, cannot be ruled out (Torres et al., 2020).

#### Biochemical Changes Associated With Sunburn Browning and Sunburn Necrosis Damage

When the combined capacity of ROS scavenging systems is exceeded and the damage incurred by ROS is not repaired between exposure times, thermal, and photooxidative damage and sunburn occur (Glenn and Yuri, 2013). While mild damage can be manifested as growth impairment and damage to the photosystems, chloroplasts or mitochondria, increasing ROS levels lead to pigment destruction (SB), lipid peroxidation, cellular membrane oxidative damage, and ultimately programmed cell death or necrosis (SN; Wang et al., 2009; Araújo et al., 2018).

In both grapes (Zschokke, 1930; Rustioni et al., 2014) and apples (Merzlyak et al., 2002) sunburn occurrence is accompanied by a loss of carotenoids and chlorophyll. While the total concentration of antioxidant enzymes and their products increase in response to sunburn, the ratios of reduced ascorbate/total ascorbate and reduced glutathione/total glutathione decrease linearly as sunlight and thermal-induced stress continue (Torres et al., 2006; Chen et al., 2008). During the photodestruction of the photosynthetic pigments, the antioxidant defense of the cells seems to be overwhelmed, and complete depletion of antioxidants (ascorbate and glutathione) ensues (Rustioni et al., 2020). When antioxidants are depleted, phenols oxidized to quinones by enzymatic (PPO and POX) or non-enzymatic reactions (ROS, autoxidation) can no longer be reduced and may polymerize to brown or black pigments (SB), possibly including non-phenolic substrates. The nature of this process and its end products have not yet been fully elucidated (Pourcel et al., 2007).

Under prolonged or extreme exposure to oxidative stress, irreversible damage occurs to the epidermal and sub-epidermal cells, which ultimately leads to thylakoid membrane destruction, cell death, and SN (Thompson et al., 1987). While relative electrolyte conductivity of cell membranes is not affected in SB fruit, it increases significantly in SN fruit, indicating the destruction of membrane integrity. This is likely caused by the initiation of lipid peroxidation, which finally leads to cell decompartmentalization and exposure of anthocyanins to ROS and consequent bleaching (Edgley et al., 2019). In addition, polyphenols are exposed to PPO and POX activity (Pourcel et al., 2007), leading to the formation of brown pigments as observed in SN. Skin cracking in grapes is accelerated by Fenton reaction catalysts (Chang et al., 2017), indicating that HO· may be involved in the skin cracking phenomena accompanying SN development.

## Factors Affecting Susceptibility

### Biotic Factors

#### Cultivar

The ability to tolerate light and heat stress varies greatly amongst individual grapevine cultivars (Silvestre et al., 2019). There is evidence from apples, but not from grapes, that sunburn susceptibility of individual cultivars may be related to fruit composition. In apples, anthocyanin accumulation increases the tolerance to light-induced photodegradation of Chl (Merzlyak and Chivkunova, 2000) and light-induced heat stress (Li and Cheng, 2009). Anthocyanin-deficient apple cultivars susceptible to sunburn accumulate lower amounts of carotenoids upon light exposure and show a higher level of Chl degradation than more tolerant cultivars (Merzlyak et al., 2002). Although morphological adaptation to high light and heat stress does occur in grapes (Rosenquist and Morrison, 1989; Muganu et al., 2011; Verdenal et al., 2019), the morphological properties of apple cultivars were not related to their sunburn susceptibility (Racskó et al., 2005). Similarly, the extremely sunburn-sensitive grape cultivar Bacchus had similar cuticular, epidermis, and hypodermis thickness as the rather tolerant cultivar Müller-Thurgau (Alleweldt et al., 1981), rendering it likely that it is the composition of the berry skin rather than its morphology that confers cultivar resistance against sunburn. From a physical perspective, anthocyanin-containing fruit reach higher temperatures upon illumination than fruit lacking anthocyanins due to a lower albedo (Smart and Sinclair, 1976), possibly counteracting the photoprotective effects of anthocyanins. Cultivar susceptibility is also modulated by bunch morphology as tight clusters can reach higher temperatures above ambient than looser ones and large berries might reach higher temperatures than smaller ones (Smart and Sinclair, 1976).

Rustioni et al. (2015) compared the sunburn susceptibility of 20 white cultivars by exposing detached berries to artificial lighting (LED) after epicuticular wax removal. These authors classified white cultivars on a scale ranging from highly susceptible (e.g., Cornichon blanc, Riesling, Muscat of Alexandria) to tolerant (e.g., Moscato Giallo, Chardonnay, Sauvignon Blanc), based on their ability to protect Chl from photodegradation. More recently, Silvestre et al. (2019) evaluated the incidence of sunburn in 189 grapevine varieties following a heatwave in August 2018 in Alentejo (Portugal). Amongst red varieties, Alicante Bouchet, Petit Verdot, Dolcetto, Syrah, and Malbec were the cultivars that sustained the most damage whilst Touriga Franca, Touriga Nacional, Grenache, Cabernet Franc, and Cinsaut were classified as tolerant to sunburn. The only international white variety that sustained severe damage was Alvarinho, and in general, white cultivars seemed to be less affected by sunburn than reds, possibly due to different vineyard management approaches. Webb et al. (2010) found no difference in sunburn incidence between red and white cultivars; and reported the most severe damage for Viognier, Pinot Noir, Semillon, and Shiraz; while Grenache, Pinot Gris, and Sauvignon Blanc were the least affected. These rankings of susceptibility under field conditions disagree with the browning index proposed by Rustioni et al. (2015) for some varieties. These discrepancies might be explained by the different approaches taken by the authors, i.e., surveying damage in the field and exposing formerly shaded, detached berries to high-light conditions in the lab. Additionally, the comparison of these results is complicated by a lack of common scale for sunburn damage determination and by the high influence of meta-data, such as cumulative temperatures, water status, UV-B radiation irrigation, and cultural practice.

Likewise, sunburn susceptibility in table grape varieties appears to be unrelated to berry color, with varieties like Calmeria (green berries) and red globe (red berries) being classified as highly susceptible whilst Italia (golden berries) and Flame seedless (red berries) have a low susceptibility (Hannah et al., 2002). Breeding strategies for table grape varieties have developed in different directions than wine grapes, as different characteristics (i.e., visual attributes and sugar loading capacity) have been prioritized for each of these crops. Amongst the characteristics prized in table grapes is their ability to maintain turgor, cultivar selection has thus made them less susceptible to shrivel than wine grapes (Hannah et al., 2002).

#### Developmental Stage

Contrasting findings have been reported regarding the influence of developmental stages on sunburn susceptibility. Hulands et al. (2014) reported that grape berry susceptibility to sunburn seems to be lowest at the early stages of berry development, and increasing thereafter. They found no significant effects of a high light/high temperature treatment on berry composition and sunburn incidence when Semillon berries were treated early (berry size ~7 mm), whereas the same conditions were found to significantly affect sunburn damage at later stages of development (Hulands et al., 2013, 2014). These findings are supported by Webb et al. (2010), who reported low sunburn damage in pre-véraison grapes and the highest damage during véraison. In contrast, Gouot et al. (2019a,b) have reported higher thermal susceptibility earlier in the season, with tissue necrosis occurring from FST 44.8°C at EL-31 (pea size, Coombe, 1995) and only from 50°C after véraison in Shiraz berries. This is consistent with results from Müller-Thurgau (1883), who reported damage thresholds of 43°C for pre-véraison berries and 55°C for ripening berries of different cultivars. Further, pre-véraison SN symptoms appear in a matter of hours after treatment (Zschokke, 1930) while SN occurring during ripening leads to much slower shriveling and longer delay times (up to 5 days) for the appearance of symptoms (Nuzzo et al., 2009). Post-véraison SN often also leads to a lower loss in yield when compared to pre-véraison SN.

The varying susceptibility during berry development may relate to a very high ratio of photoprotective pigments to chlorophylls during and shortly after flowering, which gradually decreases during berry development. The concentration of many berry skin pigments and antioxidants on a surface area basis seems to be at a maximum (as is the capacity to up-regulate their biosynthesis) shortly after flowering and decreases thereafter. This has been shown for Chl a and b, a variety of carotenoids including those from the xanthophyll cycle, and berry skin phenolic compounds. The ratio between NPQ and electron transport rate in Kerner and Portugieser also seemed to be at a maximum shortly after flowering (Düring and Davtyan, 2002). During the early stages of development, chloroplasts are still active and berry behavior is more akin to that of leaves, which have developed a series of photoprotective mechanisms to protect the photosynthetic apparatus; a capacity that is progressively lost as berries develop (Joubert et al., 2016). Downey et al. (2004) showed that Chl concentration in berry skins of Shiraz decreased constantly after flowering, accompanied by a decrease in berry skin flavonol and tannin concentration, as well as *FLS* expression. Only after véraison, *FLS* expression and flavonol concentration reaches levels comparable to the flowering stage (Downey et al., 2003). Similarly, carotenoid concentration and waxes (on a surface area basis), as well as the activity of several antioxidant enzymes of grape berries seems to decrease from pea-size towards ripening (Kwasniewski et al., 2010; Muganu et al., 2011; Joubert et al., 2016). These observations might explain why early defoliations (around flowering) have been shown to be more efficient at decreasing susceptibility to sunburn when compared to defoliations performed at pea size and véraison (Gambetta et al., 2019b; Verdenal et al., 2019). At véraison, sunburn protection in grape berries appears to change from a chloroplast-based defense strategy mediated by carotenoids to a strategy based on the accumulation of phenolics, as well as ascorbate (Melino et al., 2009) and GSH (Adams and Liyanage, 1993) in their respective reduced forms. Grape susceptibility to sunburn is thus likely to peak around véraison, when the concentrations of anthocyanins and/or flavonols, ascorbate and GSH, as well as the Car/Chl ratio are comparatively low. Véraison also coincides with the initiation of the second phase of berry expansion that is likely accompanied by ROS-mediated cell wall softening. A study on loquats subjected to high-light and high-temperature regimes at different points in ripening (green, color-changing, and yellow) have also demonstrated differences in the level of expression of the main ROS scavenging enzymes between different ripening stages. Loquats appear to be particularly susceptible to sunburn when changing color from green to yellow (a developmental stage similar to véraison in grapes), with glutathione peroxidase levels at their lowest during color change and dehydroascorbate reductase expression decreasing as the fruit ripened (Jiang et al., 2015).

### Abiotic Factors

#### Water Status and Transpiration

A sufficient water supply promotes canopy transpiration throughout much of the day, lowering the temperature and increasing the relative humidity (RH) in the bunch zone. Consequently, lower canopy transpiration under drought stress might increase FST and sunburn risk (Tarara and Spayd, 2005). Berry transpiration directly reduces FST, making it a potentially important contributor to sunburn protection. Müller-Thurgau (1883) sought to demonstrate this in an early experiment: when he heated berries in dry air (high transpiration), sunburn symptoms appeared at an air temperature of 44°C, while berries heated in water-saturated air (no transpiration) showed symptoms at 41.5°C. However, berry transpiration correlates linearly with VPD, as grape berries lack the ability to regulate transpiration (and thus, FST) actively (Zhang and Keller, 2015). Further, berries cut from drought-stressed vines transpired similar amounts of water as those cut from well-watered vines (Dimopoulos et al., 2020). Therefore, it is unlikely that water status influences sunburn incidence *via* berry transpiration.

Drought stress promotes ROS production in plants by increased electron leakage from PSII to the Mehler reaction and increased photorespiration. In most species, ROS homeostasis under drought is maintained by an increase in antioxidative defense (e.g., SOD, APX, GR) but when the capacity to scavenge ROS, is overwhelmed during prolonged or severe drought stress, oxidative damage occurs, ultimately leading to cell death (Cruz de Carvalho, 2008). In grape berries, limited water supply increases the incidence of cell death when compared to the effects of high light and temperatures on their own (Carvalho et al., 2016). However, drought stress priming has also been shown to promote resistance to heat stress *via* cross-priming reactions in wheat (Wang et al., 2015), and cross-talk between the response to both stresses has been reported in grapevines (Rocheta et al., 2014). This is not surprising, as the antioxidative systems stimulated by drought stress are general ROS defense mechanisms. It was recently demonstrated that grapes from drought-stressed vines also accumulate higher amounts of epicuticular wax than grapes from non-stressed vines (Dimopoulos et al., 2020), potentially increasing resistance to high-light conditions.

Finally, drought stress leads to reduced vigor and smaller canopies which increase bunch exposure and the potential damage by sunburn inducing conditions. Fruit from vigor-constrained drought-stressed canopies are, however, better acclimated to light and heat, and are therefore less sensitive than fruit from dense canopies that are suddenly exposed by cultural practices like leaf removal or hedging.

#### Wind

Sunburn appears to occur less frequently under windy conditions, mostly due to its cooling effect *via* forced convection, but also to increased berry transpiration at higher wind velocities. FST on the “hot spot” of a fully irradiated ripe berry is 5°C lower when wind velocity increases from 0.5 to 2.0 m·s^−1^ (Smart and Sinclair, 1976). As direct sunlight elevates berry temperatures above air temperature, forced convection inevitably cools down sun-exposed berries. Although some authors have held that windy conditions might play a role in sunburn phenomena by substantially increasing berry transpiration, ultimately leading to a hydraulic failure (Schultz, 2007), there is no experimental evidence for this hypothesis. In general, as wind velocity increases, sunburn incidence decreases (Racskó and Schrader, 2012).

### Management Practices and Vineyard Layout

Many viticultural management practices directly affect fruit sunlight exposure and therefore, sunburn incidence. An additional consideration is the crop load, closely related to pruning level and the number of buds retained, as it also influences bunch exposure. Worse sunburn damage has been observed when canopies are small and crop loads high (Dry, 2009).

#### Leaf Removal

Practices such as defoliation are intended to improve aeration, spray penetration, and berry coloration (in red varieties) and decrease disease pressure, but when performed inadequately can lead to a higher canopy porosity increasing the percentage of sunburn. Commonly performed in cool and moderate climates where fruit maturation can be difficult or disease pressure high, the increase in heatwave frequency has made this practice problematic in hot or Mediterranean climates.

Early defoliations (around flowering) have been shown to decrease susceptibility to sunburn when compared to defoliations performed at véraison by promoting a higher accumulation of photoprotectants when compared to defoliations performed at véraison or to non-defoliated controls (Pastore et al., 2013; Young et al., 2016; Brandt et al., 2019; Gambetta et al., 2019a; Verdenal et al., 2019). A study of the transcriptome of Sangiovese berries defoliated at different developmental stages (pre-bloom and véraison), showed that such treatments, when performed early, up-regulated genes related to the synthesis of HSPs and to the phenylpropanoid/flavonoid pathway that controls flavonol glycosylation (Pastore et al., 2013; Zenoni et al., 2017). Conversely, when defoliation was performed at véraison, the affected genes belonged exclusively to the response to stress category, indicating that leaf removal at this stage induces berry stress responses rather than adaptation mechanisms (Pastore et al., 2013).

#### Row Orientation

**Figure 9:**
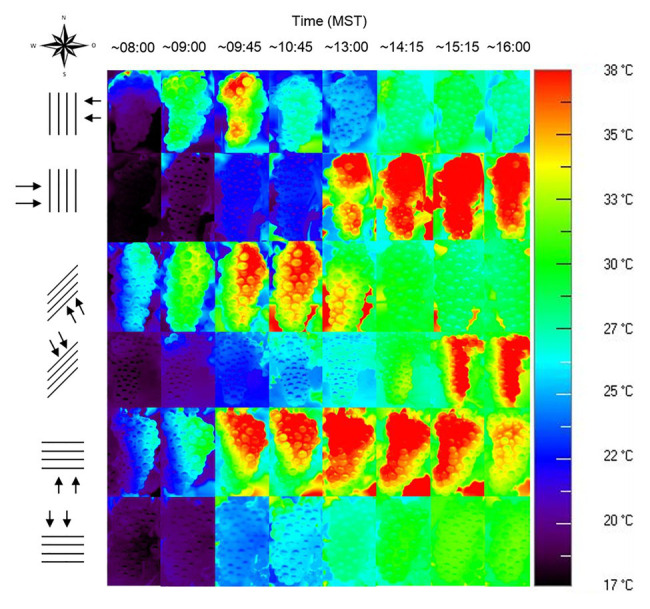
Thermal images of Riesling bunches on the two canopy sides of N-S, NE-SW, and E-W row orientations during the course of the day, taken on August 26, 2012, in Geisenheim, Germany.

Row orientation is an often-underestimated driver of sunburn, even in hot climates like Australia that have experienced substantial sunburn damage for decades. In many viticultural regions, the prevailing row orientation is N-S, which is intended to equally distribute radiation on both sides. However, while the light is indeed distributed equally between both sides of the canopy, berry temperatures differ massively between canopy sides, as E facing fruit is sun-exposed during the cool morning hours, while W facing fruit is sun-exposed during the daily maximum temperatures. At the same time, transpiration of the plant is reduced to a minimum even under well-watered conditions. In a study on Merlot grapes, west exposed berries spent an average of 70.5 h at temperatures above 35°C and 2.7 h above 40°C whilst east exposed bunches only spent 5.4 and 0 h at each of those temperatures. These differences led to sunburn symptoms being observed only on west exposed clusters (Spayd et al., 2002). Other row orientations than N-S have an unequal light distribution between canopy sides but show lower maximum bunch temperatures. In the Southern hemisphere, bunches located on the western side of an N-S oriented row spend the longest time at critical temperatures when compared to other orientations (E-W, NW-SE, NE-SW) and sides of the canopy, followed by berries on the north side of E-W rows (Dry, 2009). Bunches from the sun-exposed side of E-W oriented canopies in Germany have the highest mean temperatures and are sun-exposed during most of the day, but are shaded when ambient temperatures reach a maximum in the afternoon. Comparison to vines in N-S oriented rows within the same experiment demonstrated a higher sunburn incidence on bunches located on the W side of N-S oriented rows than on those from either side of E-W oriented rows (Figure 9). This is in accordance with an Australian survey conducted after the 2008 heatwave, which found the highest sunburn incidence occurred in N-S oriented vineyards, in which the median damage was twice as high as in E-W oriented vineyards (Webb et al., 2010). While this might be easily explained by the temperature regime, it is also worth noting that bunches on the sun-exposed side of E-W seemed to be better adapted to high light conditions, showing higher concentrations of flavonols compared to the W side of N-S oriented canopies, as they received a higher amount of radiation during the day (Friedel et al., 2016). Thus, E-W and NW-SE orientations have been recommended as a better alternative to lower FST in vineyards located in the Southern hemisphere (Dry, 2009; Webb et al., 2010). Light distribution can be further modified by row width. Narrower rows and higher canopy height create shading from neighboring plants and have been observed to decrease sunburn incidence (Danenberg, 2019).

#### Trellis and Training System

Many of the training systems that are utilized in traditional southern European and middle-eastern viticulture were developed to provide a certain degree of shelter to the grapes (e.g., gobelet, pergola). In contrast, traditional training systems in central Europe were usually designed to provide higher fruit exposure. Consequently, trellis systems that are designed to increase fruit exposure such as vertical shoot positioned (VSP), also risk overexposure of clusters (Dry, 2009). Although VSP is a popular system in many viticultural areas because of ease of mechanization, it can also increase the potential for sunburn damage. This seems to be aggravated by high bunch and berry weights normally occurring in strongly pruned systems. Alternative trellising systems such as single high-wire cordon (sprawl); head-training; tendone; pergola; Geneva Double Curtain; closing Y-shaped trellis have been proposed as suitable alternatives since they maintain bunches under a diffuse light regime and decrease direct radiation (Palliotti et al., 2014). Minimal pruning systems employed in the hottest winegrowing regions, normally also offer sufficient shelter to protect grapes from sunburn.

#### Soil and Irrigation Management

Depending on the type of soil, vineyard floor management can be an additional factor contributing to sunburn development. Bare soils reflect more light and heat than cultivated ones, especially when dealing with reflective soils such as pale-colored sands and shale (Webb et al., 2010). However, the use of cover crops can exacerbate water stress and have negative effects on canopy size by competing with vines for water. Studies have assessed the possibility of using organic (e.g., compost, bark, or straw) or synthetic (e.g., black polyethylene or geotextile) mulches instead. Less damage was observed in 2009 in Australia in vineyards with mulch and/or mown sward than in vineyards with bare soils (Dry, 2009; Webb et al., 2010).

Increasing irrigation to fill the profile has also been recommended in order to maintain the existing canopy and avoid leaf scorching and consequent fruit overexposure in the advent of heatwaves. However, a large grower survey conducted in Australia did not find any significant impacts of irrigation on sunburn appearance (Webb et al., 2010), although the authors strongly suggested irrigation as a means to prevent sunburn by maintaining canopy vitality as discussed in section Water Status and Transpiration.

## Strategies of Sunburn Protection

A number of active sunburn protection strategies are currently available on the market, including the use of netting, particle-film forming products, antitranspirants, and hydrocooling. These can be deployed as needed to mitigate damage by heatwaves or to adapt established vineyards to changing climatic conditions. Once a sunburn event has occurred, it might still be helpful to apply protective measures to prevent the spread of sunburn symptoms, especially if adverse meteorological conditions persist. This might reduce damage to berries in the cluster interior that are suddenly exposed to sunlight by the shriveling of exterior berries and might also prevent damage to the rachis.

### Netting

The most efficient way to protect grapes against sunburn seems to be the use of nets, a technique that reduces sunburn effectively in table grapes, apples, and other crops. Commercial nets range in light transmission between 20 and 70% (Briassoulis et al., 2007) and are characterized in terms of their shading factor, which depends on the net color, mesh size, and texture (Castellano et al., 2008). Depending on the type and color of netting, reductions in sunlight intensity of 4–9% (PAR), 25–29% (UV), and 5% (IR) have been measured, reducing FST by 7°C and substantially decreasing sunburn incidence (Olivares-Soto et al., 2020). Lobos et al. (2015) observed a 36% reduction in sunburn severity (termed “berry dehydration”) and FST by 7°C when using 35% shading nets, and Oliveira et al. (2014), observed a 50% decrease of shriveled berries under bunch-zone netting. While yield increased in their trial, pH and anthocyanin concentration were significantly lower in berries grown under shade nets (Oliveira et al., 2014). Contrarily, Martínez-Lüscher et al. (2017) found a significant increase in anthocyanins in netted Cabernet Sauvignon grapes when compared to the non-netted control. As berry temperature, PAR and UV radiation are simultaneously reduced by netting, this strategy seems equally effective against SB and SN.

The choice of net color seems to be as important as the type of net. Nets of different colors (e.g., red, blue, pearl, etc.) also known as photo-selective nets, scatter light, alter spectral composition and absorb different spectral bands, thus affecting grape composition and shoot and fruit growth. Peaks in the absorption spectra of cryptochromes and phytochromes have been observed in the blue and red wavelength regions and irradiation at these wavelengths have been observed to increase phenolic compounds (González et al., 2015). Green and red netting transmit 3% more green and red light respectively, and blue nets have on average a 10% higher transmittance in the blue region than black nets (Martínez-Lüscher et al., 2017; Olivares-Soto et al., 2020). When compared to pearl-colored nets, red nets were more effective at reducing sunburn incidence in apples. They provided higher protection from UV-A, and by significantly decreasing the blue/red and blue/far-red ratios, promoted a higher synthesis of anthocyanins whilst pearl-colored nets decreased their synthesis (Olivares-Soto et al., 2020). Black nets have been proven to be more effective to reduce sunburn than white nets as they provide the highest reduction in light transmission and FST whilst not modifying the spectral quality of radiation (Martínez-Lüscher et al., 2017; Manja and Aoun, 2019). Black nets also preserved total anthocyanins more, and anthocyanins and flavonols exhibited higher hydroxylation levels than those under other net colors (blue, pearl, aluminet; Martínez-Lüscher et al., 2017).

### Particle Film Forming and Antitranspirant Products

Chemical reflectants such as kaolin and calcium carbonate (CaCO_3_) have been trialed with success in different fruit crops. Kaolin [Al_2_Si_2_O_5_(OH)_4_] is an inert white clay that can reflect UV and IR and reduce FST (Rodriguez et al., 2019). Application of kaolin reduced FST by 1°C and sunburn severity by 12.5%, while fruit quality remained unchanged or even increased (Brillante et al., 2016). CaCO_3_ acts in a similar way to kaolin. In Red Roomy grapes sunburn incidence was reduced from 14.8–15% (control) to 1.7–2% when a 2% CaCO_3_ solution was applied (Ahmed et al., 2013). Results from trials on grapes, as well as on pomegranate fruit treated with kaolin have shown an increase in total polyphenols, anthocyanin, and ascorbate content (Dinis et al., 2016; Sharma et al., 2018). The application of particle films only marginally decreases FST but increases the reflection of radiation. Hence, this strategy appears to be more effective against SB than SN.

An alternative to particle film-forming products are pine resin-based products which possess antitranspirant properties. Results about the effectiveness of these products in viticulture are so far inconclusive. While Fahey and Rogiers (2019) showed that pinolene application was successful in lowering fruit transpiration, Rodriguez et al. (2019) showed that FST and sunburn actually increase due to a lack of transpiration. Further, Brillante et al. (2016) observed a decrease in fruit quality and consumer preference for the wines made with these products.

Other forms of transpiration regulation include the use of abscisic acid (S-ABA). S-ABA is a growth regulator that controls stomatal closure, transpiration, and the plant’s response to water stress. Foliar application of S-ABA has been trialed on apples in Japan and South Africa. Similar to pine-based products, results are inconclusive (Iamsub et al., 2009; Zenoni et al., 2017).

### Evaporative Cooling

This method consists of wetting the fruit and/or the canopy with overhead sprinklers or micro-sprinklers above or under the canopy in order to reduce FST and thus SN. Yang (2018) provided a detailed model for the activation of micro-sprinklers in northern highbush blueberries to avoid sunburn damage. Greenspan (2009) reported that under-canopy and over-canopy cooling using micro-sprinklers reduced FST by 5°C and almost 12°C, respectively, compared to control vines, reducing sunburn, and berry dehydration.

### Bagging

Fruit bagging is often used to produce high-quality table grapes, enabling a good and homogenous coloration, aromatic quality, and protection against grape berry moth and sunburn (Karajeh, 2018). Paper bags have been cited as being as effective as dark nets in reducing sunburn (Tsai et al., 2013); they reduce the temperature inside the bag and block direct sunlight, which makes them effective against both SB and SN. The efficiency of bags depends on the color and material, as several options exist.

## Consequences for Fruit Quality and Winemaking

**Figure 10:**
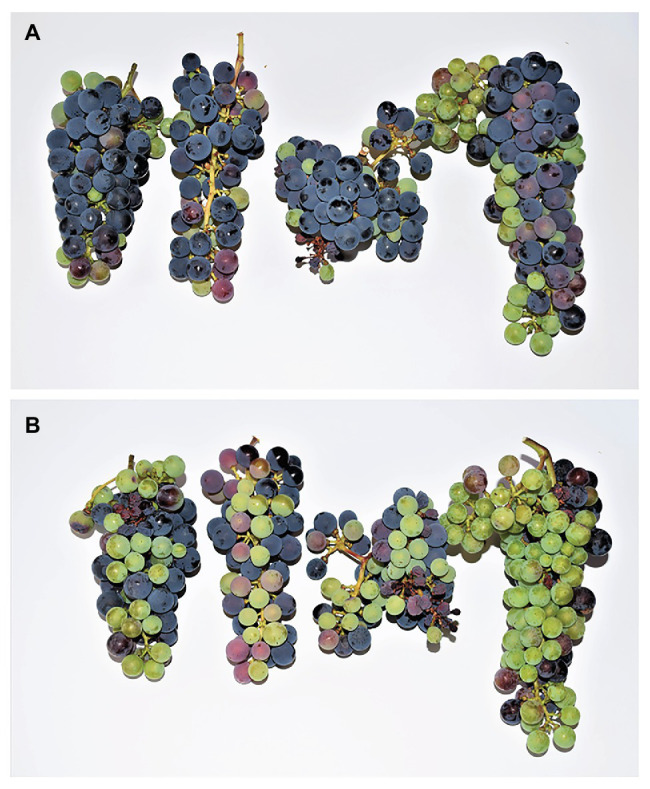
Uneven berry development induced by light and heat overexposure in Cabernet Sauvignon with minimal sunburn damage (0–3%). **(A)** Eastern side of four bunches showing normal development. **(B)** Western side of the same bunches, showing delayed color change, smaller berries (mean: 1.04 vs. 1.29 g), and a delayed sugar accumulation (mean: 8.7 vs. 15.2°Brix). Images were taken on August 24, 2020, in Geisenheim, Germany, after a pre-véraison heatwave that occurred from August 7, 2020 to August 12, 2020.

Whereas SN leads to shriveled berries and mostly impacts yields, SB affects berry composition with a consequent detrimental effect on wine quality. It is often unclear, however, whether the negative impact on wine sensory characteristics results from the sunburnt berries themselves or if it is simply a consequence of fruit overexposure to heat and sunlight. A study by Bondada and Keller (2012) on Cabernet Sauvignon berries showed lower TSS, tartaric, and malic acid levels in berries affected by sunburn when compared to healthy berries. The observed lower levels of tartaric and malic acid, however, were probably due to temperature-induced degradation rather than sunburn itself (Tarara and Spayd, 2005; Pastore et al., 2013; Brandt et al., 2019). The effect of SB on TSS is not clear, with multiple studies reporting inconsistent results across vintages or no effect at all on this parameter (Spayd et al., 2002; Greer and La Borde, 2006). This seems logical, as SB is mostly a skin phenomenon with little to no effect on the pulp. Nevertheless, uneven ripening is a disorder associated with sunburn in practice (Figure 10). Temperatures over 30°C overall flavonoid content, especially anthocyanin concentration (Pastore et al., 2013), a phenomenon likely related to oxidative stress (Mori et al., 2007). SB and SN lead to a further decrease of anthocyanin concentration, compromising wine color.

The response of aroma compounds in relation to sunburn damage has not been studied. However, studies under less stressful conditions than those leading to sunburn have demonstrated that light exposure modulates the synthesis of many compounds including the aroma compounds. Under moderate climatic conditions; increased PAR and UV radiation increased the final concentrations of terpenes; including linalool, citronellol, nerol and geraniol, and C_13_-norisoprenoids; including TDN, 3-oxo-α-ionol, β-ionone, and β-damascenone; whilst increased UV radiation decreased the amount of ethyl esters of fatty acids in Pinot Noir wine (Marais et al., 1992; Schüttler et al., 2015; Song et al., 2015; Friedel et al., 2016; Sasaki et al., 2016; Young et al., 2016; Gambetta et al., 2017). In varieties like Riesling that are prone to accumulate TDN, it is reasonable to infer that this compound could increase to values above the perception threshold, negatively impacting the aroma quality of the wine. The effect of higher temperatures depends on the aroma class in consideration; C_13_-norisoprenoid concentration is higher in grapes from warmer climates, although extreme temperature (>35°C) appears to induce their degradation (Asproudi et al., 2016; Gambetta et al., 2017). Likewise, between 20 and 40°C, the concentration of terpenes increases whilst temperatures favoring sunburn development (above 40°C) inhibit the enzymes in the mevalonate pathway, reducing terpene synthesis while also increasing their degradation (Loreto and Schnitzler, 2010). Bagged fruit and fruit shaded in boxes retained a higher amount of aromatic compounds like monoterpenes and C_6_ alcohols in hot climates (Bureau et al., 1998; Scafidi et al., 2013), indicating a degradation of aromatic quality under light and heat stress (Scafidi et al., 2013). Such conditions also impact on wine proteins and increase the tendency to form haze (Meier et al., 2016).

There are very few reports of the consequences of sunburn on wine quality. SB has been linked to undesirable phenolic characters (in particular in regards to white berries), a general loss of flavor and increased bitterness and browning of white wines (Allan, 2003; Dry, 2009). Likewise, Greer and La Borde (2006) reported increased brown coloration and bitterness in Chardonnay wines produced with sunburnt berries, and lower overall quality as reported by a sensory panel. These wines had more intense peaks at 440 nm suggesting a higher content of polyphenols that could be responsible for the increased bitterness. Red wine quality is intimately related to color, and as the appearance of sunburn symptoms requires berries to spend a certain amount of time above critical temperature thresholds (30–35°C), the consequent degradation of anthocyanins leads to a loss of coloration and ultimately color bleaching, decreasing overall wine quality (Kliewer and Torres, 1972).

Necrotic SN berries remain on the vine if harvest machines are adjusted correctly or can be removed by automated sorting tables employing airflow or density sorting processes (Lafontaine and Freund, 2013). They may, however, be problematic when present in fermentations on the skins. The modification of winemaking techniques such as lower pressing intensity and limited phenol extraction through shorter skin contact together with careful fining could be envisageable to limit the negative effects of sunburn on wine composition. More work on this topic is necessary.

## Future Perspectives

Great progress has been made in our understanding of sunburn in the past decade thanks to advancements in both analytical and molecular technologies. However, most of this knowledge has been generated on apples. Although apples and grapes share many common stress responses, differences in composition, physical properties, management and growth conditions, as well as their ability to adapt to stress make it difficult to extrapolate all findings from apples to grapes. Consequently, additional research is needed about sunburn in both wine and table grapes.

To yield comparable experimental results, future research should use a clear nomenclature of sunburn type, report severity and incidence of the damage. Access to accurate metadata, such as developmental stage, vineyard layout, fruit exposure and climatic conditions preceding the event, plant material, and cultural practice would be ideal. Information on vineyard layout and site characteristics would aid with the interpretation of SN and SB data collected in field surveys and would make large amounts of data accessible for research. Also, if provided correct metadata, an objective classification of the sunburn susceptibility of different grape varieties would be possible. This could guide producers’ choice of planting material and management practices. The comparison of susceptible and tolerant varieties on a compositional and morphological level might help to identify traits conferring tolerance to high light and temperature, which would also be of use in phenotyping new tolerant varieties and clones. If the susceptibility of a given cultivar and developmental stage and the duration of adaptation were known, this information could be combined with accurate berry FST models to predict sunburn events. In addition, modeling approaches on the canopy level could provide a better insight for mitigation strategies of sunburn protection considering plant architecture and training systems in vineyards.

Studies investigating sunburn susceptibility at different stages of berry development have produced conflicting results so far. It remains unclear whether these different results originate from the methodology used, the prevailing type of sunburn (which is often not reported), or from cultivar-specific differences. If experimental plants grown under standardized conditions were exposed to combined heat and light stress at different developmental stages, response surfaces for SB and SN could be produced with a limited set of experiments in controlled environments. This would greatly advance the current understanding of SB and SN thresholds and their physiological background. Although recent progress has allowed to discriminate between short, medium, and long term adaptation to stress, it remains unclear how long it takes the berry to become fully adapted to light and heat stress, or which conditions favor specific adaptation strategies.

Finally, although the consequences of SB and SN on the visual appearance and yield of grapes are increasingly well understood, there is very little insight into their effects on wine composition and quality, as are the oenological measures that have the best potential to alleviate sunburn-related problems. Whether it is a reduction of pressing intensity or changes in type and dosage of fining agents, understanding the best ways to manage affected fruit will help winemakers reduce economical losses at the winery as global temperatures and the incidence of sunburn rise.

## Conclusion

Sunburn is mainly a consequence of photooxidative damage that is exacerbated by thermal stress. When faced with light and/or heat stress, the berry activates a cascade of reactions aimed at protecting its photosynthetic apparatus by compensating the accumulation of toxic ROS species. This is accomplished through an increased production of antioxidants, HSPs, carotenoids, and polyphenols. It is worth noting that research on the antioxidative apparatus of fruit is far from complete, and the relative contribution of different antioxidative defense pathways is not yet fully understood. Furthermore, these responses vary with the developmental stage of the fruit, the degree of acclimation and interaction with other environmental and biological factors. When the capacity of the berries to detoxify ROS is overwhelmed, permanent changes in the visual appearance and composition of the peel occur. Under sub-lethal conditions, SB occurs while lethal conditions lead to cell death accompanied by necrosis.

As temperature and drought increase with climate change, the frequency of sunburn is set to increase. Furthermore, this problem is not restricted to a particular region but is a worldwide phenomenon that leads to non-negligible economical losses and as such, merits the study of prevention and correction measures, at the vineyard and winery level. The best prevention measures are those that achieve a reduction of both intercepted light (PAR and UV) and FST. Preventive measures in the vineyard include seasonal practices such as timing and intensity of leaf removal and hedging, irrigation including evaporative cooling and application of reflectants or nets, and long-term adaptation range from cultivars selection to structural adaptation in the vineyard such as training systems or row orientation. Information is particularly lacking on the organoleptic consequences of producing wine with sunburnt berries, and if there are any tolerance/rejection thresholds that should be considered for this type of damage. Further study could help clarify these aspects as well as develop effective corrective measure at the winery.

## Author Contributions

JG and MF initiated and designed the overall concept and wrote the manuscript. All authors revised the manuscript, approved the final version and approved it for publication.

### Conflict of Interest

The authors declare that the research was conducted in the absence of any commercial or financial relationships that could be construed as a potential conflict of interest.
